# Global, regional, and national trends in thyroid cancer burden (1990–2021): Insights from the GBD 2021 study

**DOI:** 10.17305/bb.2025.12503

**Published:** 2025-06-16

**Authors:** Ming Tang, Jiarui Li, Mingxiu Sun, Xin Song, Kaize Zheng, Xiaoting Luo, Zhirui Xue, Likun Du

**Affiliations:** 1 Heilongjiang University of Chinese Medicine, Harbin, Heilongjiang, China; 2 First Affiliated Hospital, Heilongjiang University of Chinese Medicine, Harbin, Heilongjiang, China; 3 Liaoning University of Traditional Chinese Medicine Xinglin College, Shenyang, Liaoning, China

**Keywords:** Thyroid cancer, TC, GBD database, risk factors, burden prediction

## Abstract

The global incidence of thyroid cancer (TC) has been steadily increasing and is now recognized as one of the most prevalent endocrine malignancies. This study provides a comprehensive evaluation of the prevalence, incidence, mortality, and disability-adjusted life years (DALYs) associated with TC from 1990 to 2021. Data for this study were sourced from the 2021 Global Burden of Disease (GBD), Injuries, and Risk Factors Study. To quantify temporal patterns and assess trends in age-standardized TC metrics—namely, age-standardized prevalence rate (ASPR), age-standardized incidence rate (ASIR), age-standardized death rate (ASDR), and DALYs—estimated annual percentage changes (EAPCs) were calculated. The analysis was stratified by sex, 20 age groups, 21 GBD regions, 204 countries/territories, and five Socio-demographic Index (SDI) quintiles. Statistical analyses and plotting were conducted using R statistical software version 4.4.2 and Joinpoint software. The study found that the global burden of TC remains substantial, with a significant increase in the total number of cases. In 2021, regions with high SDI reported the highest ASPR, showing an upward trend compared to 1990; however, this trend began to decline significantly after 2009. Conversely, regions with low and low-middle SDI exhibited noticeable increases in ASPR, ASIR, ASDR, and DALYs. The highest prevalence and incidence were observed in the 55–59 age group, followed by a gradual decline. The majority of affected individuals were women. A high body mass index (BMI) was identified as the primary risk factor for TC, and both prevalence and incidence are expected to continue rising through 2040.

## Introduction

The incidence of thyroid cancer (TC) continues to rise globally and ranked as the tenth most common cancer worldwide in 2020, with a higher prevalence among women [1]. The most common types of TC are papillary carcinoma (accounting for 84%, predominantly affecting women [2]), follicular carcinoma (4%, more prevalent in iodine-deficient regions), and anaplastic carcinoma (the most aggressive type, associated with a poor prognosis [3]). Most well-differentiated TCs are asymptomatic and are often detected incidentally during routine physical examinations or imaging studies. However, they may present with compressive symptoms such as hoarseness, dysphagia, and dyspnea. Epidemiological studies on TC have produced the following findings: Bao et al. [4] observed a continuous global increase in TC incidence and identified high body mass index (BMI) as a significant risk factor for the disease. Between 2000 and 2019, the overall incidence rate in the United States was approximately 13.22 cases per 100,000 individuals [5]. Asia accounts for over 50% of global TC cases, with Southeast and East Asia being particularly affected [6]. Incidence and mortality rates in Eastern Europe are significantly higher than in Western Europe [1]. Epidemiological studies have consistently highlighted persistent disparities in disease prevalence and incidence across geographic regions and different quintiles of the Socio-demographic Index (SDI). These variations significantly influence the global burden of TC. However, no comprehensive analysis has yet been conducted using the most recent Global Burden of Disease (GBD) 2021 data to assess the worldwide burden of TC. To address this gap, we aim to provide an updated assessment of global, regional, and national trends in TC prevalence, incidence, mortality, and disability-adjusted life years (DALYs) from 1990 to 2021. Our analysis is stratified by sex, age, and SDI to identify the populations most affected by TC. Additionally, we incorporate an analysis of risk factors to inform targeted prevention and treatment strategies.

## Materials and methods

### Data collection

The GBD 2021 study provides a comprehensive assessment of health losses attributable to 371 diseases, injuries, and disabilities, as well as 88 risk factors, across 204 countries and regions. This study utilized [7] the most up-to-date epidemiological data and standardized methodologies, improving upon previous iterations by stratifying the data by country, age, year of occurrence, and sex [8]. Detailed information on the study design and methodology has been extensively documented in the existing GBD literature [9]. The study collected data on the number and rates of prevalence, incidence, deaths, and DALYs, along with SDI and population data across different age groups, from 21 GBD regions worldwide. These regions are categorized based on socioeconomic similarities and geographic proximity, including Andean Latin America, Australasia, North Africa and the Middle East, Oceania, Eastern Sub-Saharan Africa, Southeast Asia, Eastern Europe, East Asia, High-income North America, High-income Asia Pacific, the Caribbean, and others.

### Estimation framework

Incidence and prevalence rates were calculated using DisMod-MR 2.1 (Disease Model – Bayesian Meta-Regression). Mortality estimates were derived using the CODEm framework, which integrates vital registration and verbal autopsy data that are rigorously adjusted prior to analysis to ensure accuracy. CODEm improves the precision of estimates by incorporating multiple models and accounting for variations in study design and methodology across data sources. This approach enhances both the consistency and reliability of the final estimates. To calculate DALYs due to TC, two components are summed: years lived with disability reflecting the impact of surviving TC, and years of life lost due to premature mortality. In GBD 2021, countries/regions are classified into five tiers based on SDI, which is quantified using fertility rates, education levels, and per capita income: high SDI (>0.81), high-middle SDI (0.71 < SDI ≤ 0.81), middle SDI (0.61 < SDI ≤ 0.71), low-middle SDI (0.46 < SDI ≤ 0.61), and low SDI (SDI ≤ 0.46). The SDI ranges from 0 to 1, with higher values indicating higher levels of development [10].

### Risk factors

We focused on the risk factor of high BMI as identified in the GBD 2021 study. The data on DALYs and mortality from TC attributable to this factor were analyzed and stratified by region to reveal geographic variations in its impact. To quantify the influence of risk factors, we employed advanced modeling techniques, including DisMod-MR 2.1 and spatiotemporal Gaussian process regression, to estimate exposure distributions across populations and regions [11]. Based on epidemiological evidence, we then established the theoretical minimum risk exposure level (TMREL) for each risk factor, representing the level of exposure associated with the lowest risk of TC [12]. Using the exposure data, relative risk estimates, and TMREL values, we calculated the population attributable fractions (PAFs) for each risk factor. These PAFs were further stratified by region, age, sex, and year to quantify the potential reduction in TC burden if exposure to a given risk factor were reduced to its TMREL. We then multiplied the PAFs by DALYs to estimate the burden attributable to each risk factor. This approach highlights the potential impact of specific risk factors on TC outcomes and offers a foundation for targeted intervention strategies.

### Ethical statement

Since this study used publicly available data that did not include any confidential or personally identifiable patient information, the ethics committee granted it an exemption. Informed consent was therefore not required.

### Statistical analysis

#### Estimated annual percentage change (EAPC) regression model

To assess trends in ASR of TC prevalence, incidence, deaths, and DALYs, this study utilized the EAPC. The ASR is calculated per 100,000 individuals using the following formula: (1)
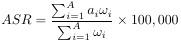


In the formula, *a*_*K*_ represents the age-specific rate for a given age group, ω_*K*_ denotes the number of individuals in the standard population corresponding to that age group, and A indicates the total number of age groups. The calculation of EAPC is based on a regression model that describes the temporal trend of ASR over a specific period. The regression formula used is: *Y* ═ *α* + *βX* + *e*, where *Y* is the natural logarithm of the ASR, *X* is the calendar year, *α* is the intercept, *β* is the slope (indicating trend), and *e* is the error term. The formula for calculating EAPC is: 100 × [exp(β) − 1]. The 95% CI for the EAPC is calculated using a linear regression model. If both the EAPC and the lower bound of its 95% CI are positive, the ASR is considered to show an upward trend. If both are negative, it indicates a downward trend. If neither condition is met, the ASR is considered stable. The Spearman correlation coefficient is used to assess the relationship between DI and ASR.

### Joinpoint regression model

This study used the Joinpoint regression model to analyze trends in age-standardized prevalence rate (ASPR) from 1990 to 2021, calculating the annual percentage change (APC) and average annual percentage change (AAPC). The model identifies inflection points in disease burden trends and assesses their statistical significance [13]. If the 95% CI for the APC includes 0, the change is considered not significant. If the APC is greater than 0 and the CI does not include 0, ASPR is increasing annually; if the APC is less than 0, ASPR is decreasing annually. In the absence of inflection points, the AAPC represents a single overall trend.

### BAPC model

This study applied the Bayesian age-period-cohort (BAPC) model, using a second-order random walk to smooth the priors for age, period, and cohort effects. The model employs the integrated nested Laplace approximation (INLA) method to estimate the marginal posterior distribution and predict future trends in disease burden [14, 15].

All analyses and visualizations were conducted using the WHO’s Health Equity Assessment Toolkit and R statistical software (version 4.4.2), with Joinpoint software used to analyze trends in ASR. All statistical tests were two-sided, with *P* < 0.05 considered statistically significant.

## Results

### Global level

**Figure 1.**
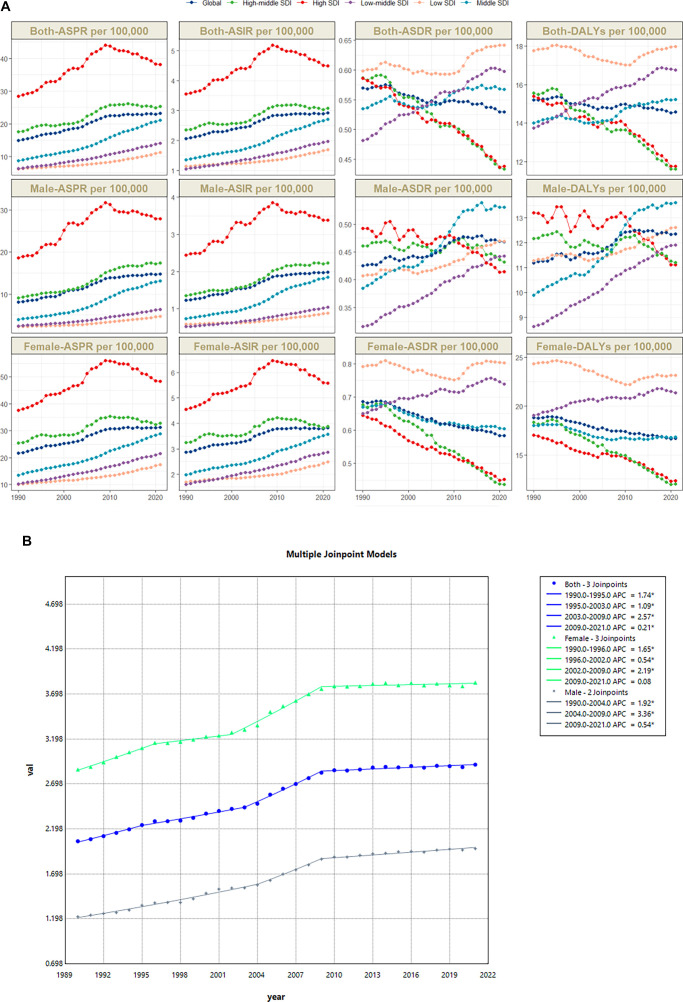
**Trend chart of TC burden from 1990 to 2021**. (A) Trends in thyroid cancer prevalence, incidence, deathes and DALYs from 1990 to 2021; (B) Age-standardization incidence rate Jointpoint regression time trend analysis. The value is followed by an asterisk (*), indicating that the change in APC is significant (*P* < 0.05). DALYs: Disability-adjusted life years; EAPC: Estimated annual percentage change; SDI: Socio-demographic index.

In 2021, the global burden of TC remained substantial, with a total of 1,987,148.5 cases (95% UI: 1,776,275.3–2,198,245.2), marking a remarkable 193.7% increase compared to 1990. Not only did the absolute number of cases rise significantly, but the ASPR also increased from 14.9 cases per 100,000 people in 1990 (95% UI: 14.1–16.0) to 23.1 cases per 100,000 people in 2021 (95% UI: 20.7–25.6). The EAPC for ASPR was 1.58 (95% CI: 1.44–1.73). In 2021, the number of new TC cases reached 249,538 (95% UI: 223,290.3–274,638.2), reflecting a 177.6% increase compared to 1990. Age-standardized incidence rate (ASIR) rose from 2.1 cases per 100,000 people in 1990 (95% UI: 2.0–2.2) to 2.9 cases per 100,000 people in 2021 (95% UI: 2.6–3.2), with an EAPC of 1.25 (95% CI: 1.14–1.37). In the same year, there were 44,798.5 TC-related deaths (95% UI: 39,924.7–48,541.0), and age-standardized death rate (ASDR) showed a slight decline, with an EAPC of −0.24 (95% CI: −0.27 to −0.21). The total number of DALYs due to TC in 2021 reached 1,246,484.8 (95% UI: 1,094,415.6–1,375,852.5), with an age-standardized DALY rate of 14.6 (95% UI: 12.8–16.1). The EAPC for DALYs was −0.14 (95% CI: −0.17 to −0.11) (Figure 1A, Table 1).

**Table 1:** Trends in TC burden: prevalence, incidence, deaths, and DALYs (1990–2021).

	**Location**	**1990**		**2021**		**EAPC_95%CI**
		**Number**	**ASR**	**Number**	**ASR**	
**Prevalence**	**Global**	**676648.8 (636788.9–727722.8)**	**14.9 (14.1–16)**	**1987148.5 (1776275.3–2198245.2)**	**23.1 (20.7–25.6)**	**1.58 (1.44 to 1.73)**
	Low SDI	21971.9 (17309–27986.9)	6.2 (4.9–7.9)	92787.3 (71789.9–126590)	11.1 (8.7–14.9)	1.87 (1.76 to 1.99)
	Low-middle SDI	56029.2 (47678.1–71121.3)	6.3 (5.3–7.9)	251682.8 (206843.3–308118.1)	14 (11.5–16.9)	2.67 (2.64 to 2.7)
	Middle SDI	122505.4 (108257.6–142672.6)	8.6 (7.7–10.1)	594136.2 (493696.7–673492)	21 (17.5–23.8)	3.03 (2.95 to 3.12)
	High-middle SDI	186568 (174097.6–198522.8)	17.6 (16.4–18.7)	448569.8 (401405.4–510752.5)	25.2 (22.6–28.8)	1.4 (1.22 to 1.58)
	High SDI	288686.4 (280129.1–297109)	28.5 (27.6–29.3)	598370 (566881.9–629719.5)	38.2 (36.3–40.4)	1.23 (0.93 to 1.53)
	Andean Latin America	2640.4 (2211.1–3138.8)	9.8 (8.1–11.5)	18047.3 (13837.2–22793)	28.1 (21.6–35.6)	3.55 (3.34 to 3.77)
	Australasia	4599.9 (4076.9–5210.9)	20.6 (18.3–23.3)	16211.2 (13090.8–19620.8)	38.9 (31.4–47.6)	2.87 (2.29 to 3.47)
	Caribbean	3118.1 (2873.6–3378.8)	10.6 (9.8–11.5)	9357.3 (8102.5–10834.1)	17.8 (15.4–20.6)	1.86 (1.69 to 2.04)
	Central Asia	6945.8 (6347.8–7593.4)	12.7 (11.6–13.9)	13021.3 (11400.3–14782.4)	13.4 (11.7–15.2)	0.16 (−0.61 to 0.93)
	Central Europe	34174.4 (32484.3–36024.9)	23.6 (22.4–24.8)	37892.2 (34073.8–41467.7)	21.9 (19.7–24)	−0.33 (−0.52 to −0.14)
	Central Latin America	11472.2 (11049.9–11916.4)	10.2 (9.8–10.6)	58071 (51803–65647.6)	21.8 (19.4–24.6)	2.39 (2.28 to 2.5)
	Central Sub-Saharan Africa	870.5 (619–1301.5)	2.6 (1.8–3.9)	3432 (2194.6–5420.7)	4 (2.5–6.3)	1.42 (1.14 to 1.7)
	East Asia	95529.4 (76898.9–113229.6)	8.5 (6.9–10.1)	411402.4 (334530–513557.1)	20.5 (16.8–25.6)	3.16 (3 to 3.33)
	Eastern Europe	50163.3 (47847.6–52997.6)	18.9 (18.1–20)	75906.4 (68504.6–84343.5)	25.9 (23.3–28.8)	1.47 (0.97 to 1.97)
	Eastern Sub-Saharan Africa	12047 (9301.5–15328.6)	9.4 (7.3–12)	48137 (34449.7–73226.7)	15.4 (11.2–23.1)	1.47 (1.29 to 1.66)
	High-income Asia Pacific	55344.6 (51670.9–60632.8)	26.9 (25.1–29.6)	110959.1 (98931.9–129334.7)	37.1 (33.2–43.8)	1.42 (0.91 to 1.93)
	High-income North America	100673.1 (97445.8–103313)	32.1 (31.1–32.9)	237732 (226609–247666.5)	45.5 (43.6–47.3)	1.23 (1.05 to 1.42)
	North Africa and Middle East	31166 (25920.4–41990)	13 (10.8–17.4)	183491.2 (151632.4–216130.2)	30.7 (25.4–36)	3.16 (2.99 to 3.33)
	Oceania	308.3 (199–418.5)	6.8 (4.5–9.3)	960.2 (576–1382.1)	8.6 (5.2–12.3)	0.59 (0.48 to 0.7)
	South Asia	53580.2 (43818.6–70783.2)	5.9 (4.9–7.8)	282509.1 (227052.9–343508.3)	15.3 (12.3–18.5)	3.24 (3.16 to 3.33)
	Southeast Asia	45506 (36198.2–51840.3)	12.7 (10.3–14.5)	206164.7 (161849.8–244210.6)	26.9 (21.1–31.8)	2.33 (2.23 to 2.43)
	Southern Latin America	6690 (5949.1–7523.9)	14.1 (12.6–15.9)	15500.3 (13535.1–17754.5)	19.7 (17.1–22.6)	1.21 (1 to 1.42)
	Southern Sub-Saharan Africa	2655.2 (2243.8–3153)	6.9 (5.8–8.2)	8000.6 (6622.7–9541.6)	10.7 (8.9–12.7)	1.8 (1.54 to 2.05)
	Tropical Latin America	9237.1 (8742.4–9743.8)	7.8 (7.4–8.2)	33082.7 (31066.3–34983.6)	12.6 (11.8–13.3)	1.32 (1.13 to 1.51)
	Western Europe	148253.3 (140945–155876.6)	31.1 (29.5–32.7)	211123.9 (193366–229245.3)	32.7 (29.9–35.5)	0.51 (0.09 to 0.94)
	Western Sub-Saharan Africa	1674 (1212.8–2106)	1.2 (0.9–1.5)	6146.3 (4496.6–8323.4)	1.8 (1.3–2.3)	1.09 (1 to 1.18)
**Incidence**	**Global**	**89885.5 (84681.3–96998.8)**	**2.1 (2–2.2)**	**249538 (223290.3–274638.2)**	**2.9 (2.6–3.2)**	**1.25 (1.14 to 1.37)**
	Low SDI	3431.3 (2759.4–4295.6)	1.1 (0.9–1.4)	12358.4 (9598.7–16514.5)	1.7 (1.3–2.2)	1.23 (1.12 to 1.34)
	Low-middle SDI	8233.9 (7035–10302.4)	1 (0.9–1.3)	33464 (27896.3–40292.7)	2 (1.7–2.3)	2.09 (2.07 to 2.12)
	Middle SDI	17155.2 (15282.6–19997.3)	1.4 (1.2–1.6)	75356.9 (62756–84674.8)	2.7 (2.3–3)	2.37 (2.28 to 2.46)
	High-middle SDI	24410.6 (22753.3–25919.8)	2.3 (2.2–2.5)	55158.1 (49518.3–62489)	3.1 (2.8–3.5)	1.05 (0.89 to 1.21)
	High SDI	36533.2 (35292–37708.3)	3.5 (3.4–3.7)	72995.8 (68514–76746.9)	4.5 (4.3–4.7)	1.01 (0.75 to 1.28)
	Andean Latin America	422.2 (354–495.4)	1.8 (1.5–2.1)	2424 (1907.2–3044.3)	3.9 (3.1–4.8)	2.6 (2.44 to 2.77)
	Australasia	585.9 (522.4–660.1)	2.6 (2.3–2.9)	1949.1 (1569.7–2343.2)	4.6 (3.7–5.5)	2.61 (2.06 to 3.16)
	Caribbean	442.8 (410.8–479.3)	1.6 (1.5–1.7)	1244.9 (1085.7–1427.4)	2.4 (2.1–2.7)	1.5 (1.32 to 1.69)
	Central Asia	914 (840.8–996.1)	1.7 (1.6–1.9)	1630.9 (1432.2–1845.3)	1.7 (1.5–2)	0.01 (−0.73 to 0.75)
	Central Europe	4654.9 (4428.6–4883.6)	3.2 (3–3.4)	4876.2 (4406.6–5323.5)	2.7 (2.4–2.9)	−0.65 (−0.86 to −0.45)
	Central Latin America	1712.2 (1651.2–1775.4)	1.7 (1.7–1.8)	7752.6 (6907.4–8701.4)	3 (2.6–3.3)	1.66 (1.52 to 1.8)
	Central Sub-Saharan Africa	153.7 (111.5–225.9)	0.6 (0.4–0.8)	495.7 (319.2–770.7)	0.7 (0.4–1.1)	0.63 (0.42 to 0.83)
	East Asia	13203.4 (10809.5–15460.8)	1.3 (1.1–1.5)	50885.2 (41562–63161.9)	2.5 (2.1–3.1)	2.43 (2.26 to 2.6)
	Eastern Europe	6467.8 (6164.2–6831)	2.4 (2.3–2.6)	9617 (8698.1–10650.3)	3.2 (2.9–3.5)	1.27 (0.8 to 1.75)
	Eastern Sub-Saharan Africa	1908.4 (1516.6–2387.8)	1.8 (1.5–2.2)	6384.4 (4629.9–9478.7)	2.4 (1.8–3.5)	0.76 (0.6 to 0.92)
	High-income Asia Pacific	6950.1 (6496.3–7654.3)	3.4 (3.2–3.8)	14277.6 (12630.3–16476.5)	4.4 (3.9–5.2)	1.15 (0.68 to 1.61)
	High-income North America	12130.3 (11695.2–12450)	3.8 (3.7–3.9)	28289.1 (26782.7–29536)	5.3 (5.1–5.5)	1.15 (0.97 to 1.33)
	North Africa and Middle East	3792 (3150.6–5143.1)	1.7 (1.4–2.3)	21222.4 (17602–24974.6)	3.7 (3.1–4.3)	2.89 (2.72 to 3.06)
	Oceania	44.6 (29.9–60.2)	1.2 (0.8–1.6)	131.1 (80.2–185.4)	1.4 (0.9–1.9)	0.33 (0.24 to 0.42)
	South Asia	7853.1 (6476.2–10284.5)	1 (0.8–1.3)	37335.6 (30262.7–44931.4)	2.1 (1.7–2.6)	2.54 (2.46 to 2.62)
	Southeast Asia	6440.6 (5205.8–7276.5)	2 (1.7–2.3)	26559 (20898.8–31183.5)	3.6 (2.9–4.2)	1.82 (1.74 to 1.9)
	Southern Latin America	1000.1 (896.4–1118.9)	2.1 (1.9–2.4)	2046.9 (1787.2–2334)	2.5 (2.2–2.9)	0.7 (0.48 to 0.92)
	Southern Sub-Saharan Africa	376.9 (317.8–447.9)	1.1 (0.9–1.3)	1116.8 (934.8–1315.9)	1.6 (1.3–1.9)	1.54 (1.3 to 1.79)
	Tropical Latin America	1371.6 (1299.4–1443.4)	1.3 (1.2–1.3)	4491.1 (4197.7–4757.9)	1.7 (1.6–1.8)	0.76 (0.6 to 0.91)
	Western Europe	19206.5 (18291.7–20159.2)	3.9 (3.7–4.1)	26004.5 (23787.6–28201.5)	3.8 (3.5–4.2)	0.28 (−0.1 to 0.67)
	Western Sub-Saharan Africa	254.3 (186.2–312.4)	0.2 (0.2–0.3)	803.8 (597.7–1068.9)	0.3 (0.2–0.3)	0.52 (0.45 to 0.58)
**Deaths**	**Global**	**21893 (20437.5–24108.1)**	**0.6 (0.5–0.6)**	**44798.5 (39924.7–48541)**	**0.5 (0.5–0.6)**	−**0.24 (**-**0.27 to** −**0.21)**
	Low SDI	1454.5 (1199.2–1778.3)	0.6 (0.5–0.7)	3392.4 (2687.9–4328.7)	0.6 (0.5–0.8)	0.19 (0.11 to 0.28)
	Low-middle SDI	3015.5 (2624–3757.1)	0.5 (0.4–0.6)	8531.2 (7410.5–9771.6)	0.6 (0.5–0.7)	0.74 (0.71 to 0.78)
	Middle SDI	5275.6 (4815.1–6144.8)	0.5 (0.5–0.6)	14666.5 (12466.8–16170.9)	0.6 (0.5–0.6)	0.21 (0.16 to 0.26)
	High-middle SDI	5609.4 (5200.9–5934.9)	0.6 (0.5–0.6)	8436.7 (7553.5–9304.5)	0.4 (0.4–0.5)	−1.03 (−1.09 to −0.97)
	High SDI	6504.9 (6098.3–6798.4)	0.6 (0.5–0.6)	9730.2 (8465.5–10437.2)	0.4 (0.4–0.5)	−0.91 (−0.96 to −0.86)
	Andean Latin America	182.3 (153.4–211.5)	0.9 (0.8–1)	641.2 (507.9–794.5)	1.1 (0.9–1.4)	0.66 (0.54 to 0.77)
	Australasia	97.6 (87.2–109.1)	0.4 (0.4–0.5)	199.1 (161.3–237.4)	0.4 (0.3–0.4)	0.14 (−0.13 to 0.41)
	Caribbean	142.5 (131.7–156)	0.6 (0.5–0.6)	321.3 (280.7–367.5)	0.6 (0.5–0.7)	0.42 (0.2 to 0.65)
	Central Asia	253.3 (235.5–274.3)	0.5 (0.5–0.6)	339.3 (301.7–378.5)	0.4 (0.4–0.5)	−0.81 (−1.43 to −0.18)
	Central Europe	1266.4 (1213.2–1316.6)	0.9 (0.8–0.9)	985.7 (898–1064.6)	0.4 (0.4–0.5)	−2.37 (−2.65 to −2.1)
	Central Latin America	635.6 (613.9–657.6)	0.8 (0.8–0.8)	1964.6 (1748.8–2165.4)	0.8 (0.7–0.9)	−0.07 (−0.26 to 0.11)
	Central Sub-Saharan Africa	78.4 (57.7–112.8)	0.4 (0.3–0.5)	182.8 (118.4–284)	0.3 (0.2–0.6)	−0.11 (−0.23 to 0.01)
	East Asia	3780.9 (3211.5–4380.1)	0.5 (0.4–0.6)	8063.8 (6456.3–9800.1)	0.4 (0.3–0.5)	−0.65 (−0.74 to −0.55)
	Eastern Europe	1349.5 (1274–1423.5)	0.5 (0.5–0.5)	1650.5 (1508.2–1807.4)	0.5 (0.4–0.5)	−0.25 (−0.6 to 0.09)
	Eastern Sub-Saharan Africa	838.7 (685.7–1015.1)	1 (0.8–1.2)	1800.9 (1337.6–2485.5)	1 (0.7–1.3)	−0.18 (−0.29 to −0.07)
	High-income Asia Pacific	1235.9 (1126.1–1404.5)	0.6 (0.6–0.7)	2843.9 (2308.9–3185.8)	0.5 (0.4–0.6)	−0.82 (−0.98 to −0.65)
	High-income North America	1391.8 (1285–1450.1)	0.4 (0.4–0.4)	2765.7 (2473–2948.9)	0.4 (0.4–0.4)	0.15 (0.06 to 0.23)
	North Africa and Middle East	657.9 (546.1–936.5)	0.4 (0.3–0.6)	1935.2 (1676.1–2236.4)	0.4 (0.4–0.5)	0.64 (0.49 to 0.79)
	Oceania	14.6 (10.3–19.3)	0.6 (0.4–0.7)	36.6 (23.3–50.4)	0.5 (0.4–0.7)	−0.13 (−0.16 to −0.1)
	South Asia	2914.5 (2454.1–3700.4)	0.5 (0.4–0.6)	9323.8 (7794.2–10741.5)	0.6 (0.5–0.7)	0.92 (0.88 to 0.97)
	Southeast Asia	2008.1 (1698.1–2303.1)	0.8 (0.7–1)	5642.9 (4564.6–6450.7)	0.9 (0.7–1)	0.32 (0.24 to 0.4)
	Southern Latin America	359 (324.7–394.6)	0.8 (0.7–0.9)	485.2 (425.8–553.7)	0.6 (0.5–0.6)	−1.01 (−1.26 to −0.77)
	Southern Sub-Saharan Africa	123.6 (102.4–148.3)	0.5 (0.4–0.6)	323.8 (264.5–372.4)	0.6 (0.5–0.7)	0.92 (0.65 to 1.18)
	Tropical Latin America	509 (480–537.2)	0.6 (0.5–0.6)	1252.8 (1142–1332)	0.5 (0.5–0.5)	−0.6 (−0.69 to −0.5)
	Western Europe	3951 (3667.4–4173.8)	0.7 (0.6–0.7)	3829.2 (3318.9–4190.8)	0.4 (0.3–0.4)	−1.7 (−1.77 to −1.64)
	Western Sub-Saharan Africa	102.7 (77.8–122.8)	0.1 (0.1–0.1)	210.4 (165.3–263.6)	0.1 (0.1–0.1)	−0.51 (−0.59 to −0.43)
**DALYs**	**Global**	**646740.5 (599118.8–717357)**	**15.2 (14.2–16.8)**	**1246484.8 (1094415.6–1375852.5)**	**14.6 (12.8–16.1)**	−**0.14 (**−**0.17 to** −**0.11)**
	Low SDI	52661.5 (43472.1–63947.7)	17.8 (14.6–21.6)	120143.3 (93672.8–157131.4)	18 (14.2–23.1)	−0.05 (−0.12 to 0.02)
	Low-middle SDI	103647.8 (90342.8–129887.4)	13.8 (12–17.1)	269914.2 (229089.1–316826.3)	16.8 (14.3–19.4)	0.67 (0.63 to 0.7)
	Middle SDI	166177 (149966.3–190691.4)	14 (12.8–16.2)	416217.6 (350751–460888.7)	15.2 (12.8–16.8)	0.28 (0.22 to 0.34)
	High-middle SDI	158942.1 (146716.9–170152.9)	15.6 (14.4–16.6)	218961.3 (196634.7–244418.1)	11.6 (10.4–13)	−0.99 (−1.05 to −0.92)
	High SDI	164381.5 (155870.6–173717.9)	15.4 (14.6–16.3)	220135.9 (201450.2–237834.8)	11.8 (10.8–12.8)	−0.77 (−0.86 to −0.68)
	Andean Latin America	5273.8 (4419.1–6186.5)	23 (19.3–27)	16689.8 (13207.7–20766.9)	27.5 (21.7–34.3)	0.54 (0.43 to 0.65)
	Australasia	2544 (2273.1–2867.8)	11 (9.9–12.5)	5041.1 (4103.8–6033.3)	10.6 (8.7–12.8)	0.51 (0.21 to 0.82)
	Caribbean	4116.6 (3769–4556.3)	14.9 (13.7–16.5)	8690.2 (7532.9–10099.5)	16.3 (14.1–19)	0.46 (0.25 to 0.68)
	Central Asia	8144.5 (7568–8817.1)	15.9 (14.7–17.2)	10272.9 (9055.5–11543.5)	11.7 (10.3–13)	−1.19 (−1.81 to −0.57)
	Central Europe	34786.3 (33224.2–36474.4)	23.5 (22.4–24.6)	23433.7 (21250.4–25475.1)	11.6 (10.5–12.7)	−2.47 (−2.76 to −2.17)
	Central Latin America	18293.1 (17666.1–18920.9)	19.5 (18.9–20.2)	52186.9 (47013.3–58083.4)	20.4 (18.4–22.7)	0 (−0.2 to 0.2)
	Central Sub-Saharan Africa	2545.3 (1889.3–3609.5)	9.4 (6.9–13.7)	5956.3 (3862.9–9187.8)	8.9 (5.8–13.9)	−0.19 (−0.31 to −0.06)
	East Asia	116582.2 (98062.3–136720.9)	12.2 (10.4–14.2)	213609.2 (173066.2–262361.7)	10.3 (8.3–12.5)	−0.56 (−0.67 to −0.46)
	Eastern Europe	37930 (35923.3–40329.4)	13.9 (13.1–14.7)	42085.6 (38218.3–46388.2)	12.9 (11.7–14.1)	−0.29 (−0.68 to 0.11)
	Eastern Sub-Saharan Africa	31046 (25044.5–38047.3)	30.1 (24.5–36.7)	66490.9 (48520.2–94804.4)	27.8 (20.6–38.4)	−0.43 (−0.55 to −0.32)
	High-income Asia Pacific	30855.9 (28505.4–35131.3)	15.4 (14.1–17.5)	50783.2 (43704.3–57571.4)	11.8 (10.5–13.7)	–0.75 (–0.99 to –0.5)
	High-income North America	37316 (34968.1–39721.4)	11.2 (10.5–12)	70641.6 (65003.7–76456.6)	12 (11–13)	0.21 (0.11 to 0.31)
	North Africa and Middle East	21944.4 (18189.4–30745.7)	11 (9.1–15.5)	65316.2 (55454.2–76496.3)	12.7 (10.9–14.8)	0.76 (0.62 to 0.9)
	Oceania	480.3 (324.9–641.5)	13.9 (9.8–18.5)	1173.6 (724.9–1641.6)	13.5 (8.6–18.6)	–0.1 (–0.14 to –0.06)
	South Asia	105302.9 (88400.4–135793.4)	14.2 (12–18.2)	302256.8 (249827.8–356789.4)	18.2 (15.1–21.4)	0.85 (0.81 to 0.88)
	Southeast Asia	62694.8 (51465.3–70374.5)	21.4 (17.9–24.3)	164547.4 (130332.7–189174.4)	23.6 (18.8–27)	0.27 (0.21 to 0.34)
	Southern Latin America	9491.8 (8618.3–10469.6)	20.3 (18.4–22.3)	11959.8 (10465.3–13594.3)	14.3 (12.5–16.2)	–1.02 (–1.28 to –0.75)
	Southern Sub-Saharan Africa	4035.3 (3398.8–4776.4)	12.4 (10.3–14.8)	10253.2 (8481–11926)	15.7 (12.8–18.1)	1 (0.72 to 1.27)
	Tropical Latin America	15021.1 (14259–15822.7)	14.8 (14–15.6)	32680.5 (30547–34741.4)	12.7 (11.8–13.5)	–0.65 (–0.75 to –0.55)
	Western Europe	94707.5 (89027.2–100543.1)	17.6 (16.6–18.8)	84464.5 (75823.1–92550.5)	10.5 (9.4–11.5)	–1.5 (–1.64 to –1.36)
	Western Sub-Saharan Africa	3628.7 (2761.6–4379.4)	3.2 (2.4–3.8)	7951.2 (6109.3–10289)	2.8 (2.2–3.5)	−0.49 (−0.56 to −0.42)

^Δ^The 95% Uncertainty Interval (95% UI) refers to a range within which the model predicts there is a 95% probability that the true value lies. These uncertainty intervals are typically calculated by considering factors such as variability in data sources, statistical uncertainty, and changes in model parameters. ASR: Age-standardized rate; CI: Confidence interval; DALYs: Disability-adjusted life years; EAPC: Estimated annual percentage change; SDI: Socio-demographic index; TC: Thyroid cancer.

**Figure 2.**
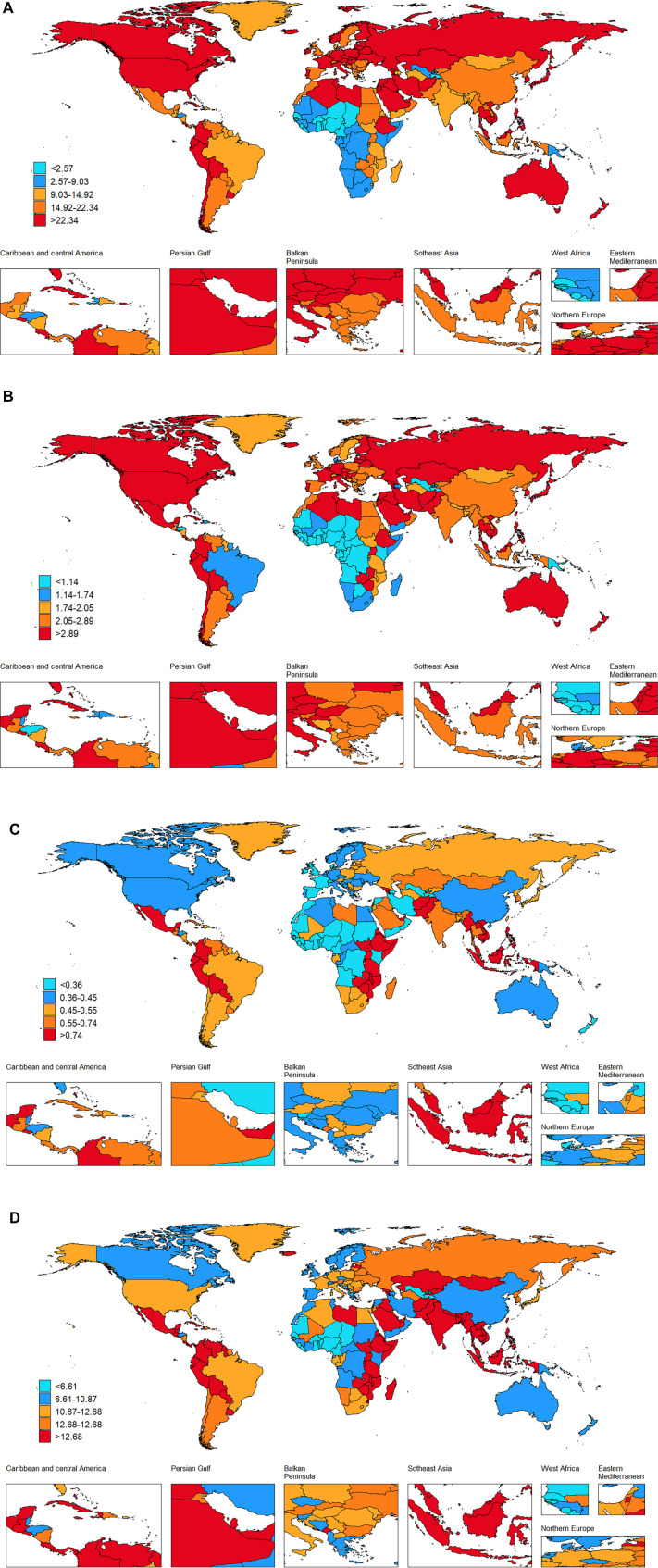
**Global disease burden in 204 countries and regions.** ^Δ^The legend in the four panels (A–D) refers to EAPC of ASR (per 100,000). EAPC: Estimated annual percentage change.

**Figure 3.**
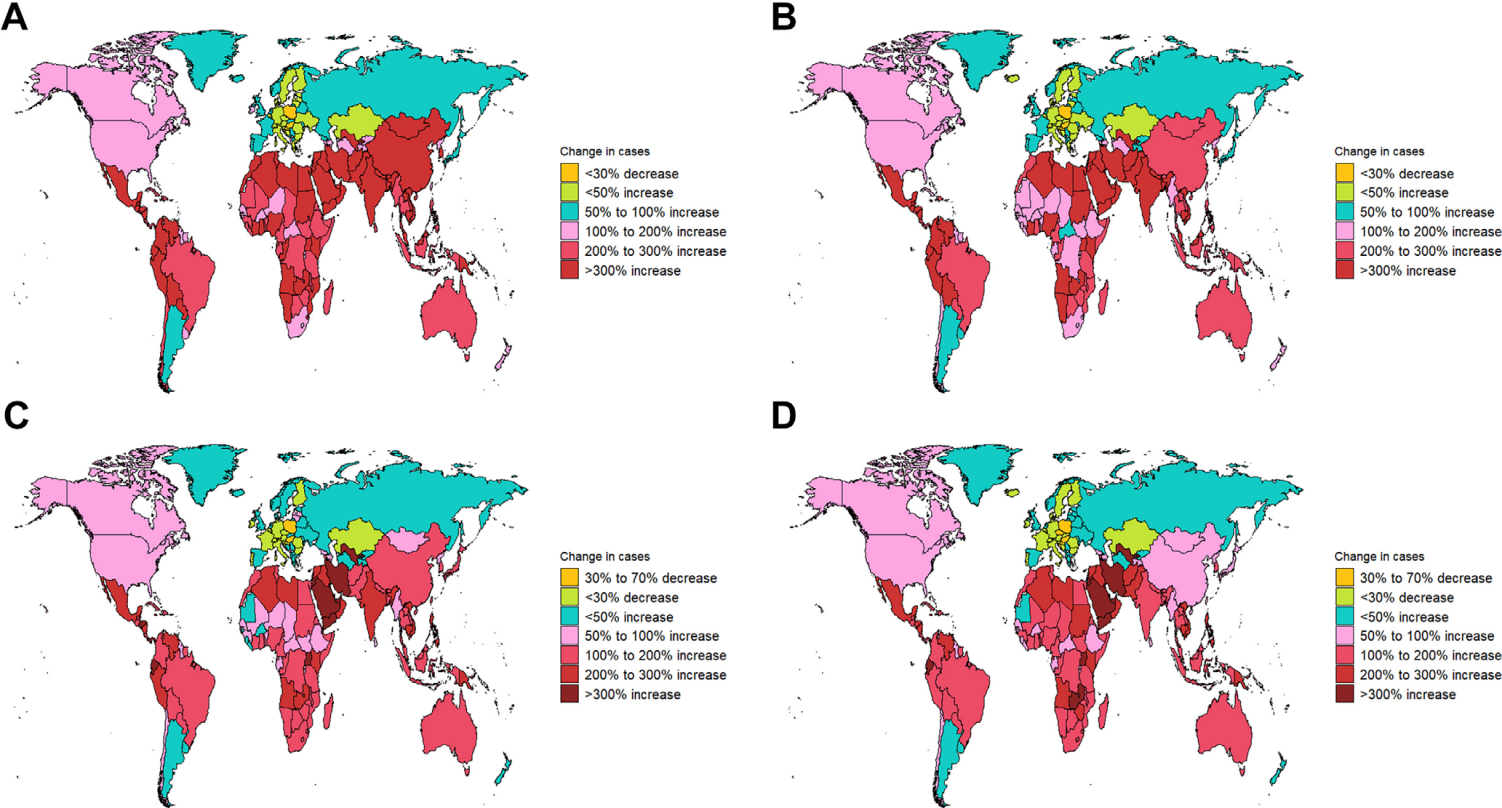
**Change cases of TC in 204 countries and territories.** TC: Thyroid cancer.

**Figure 4.**
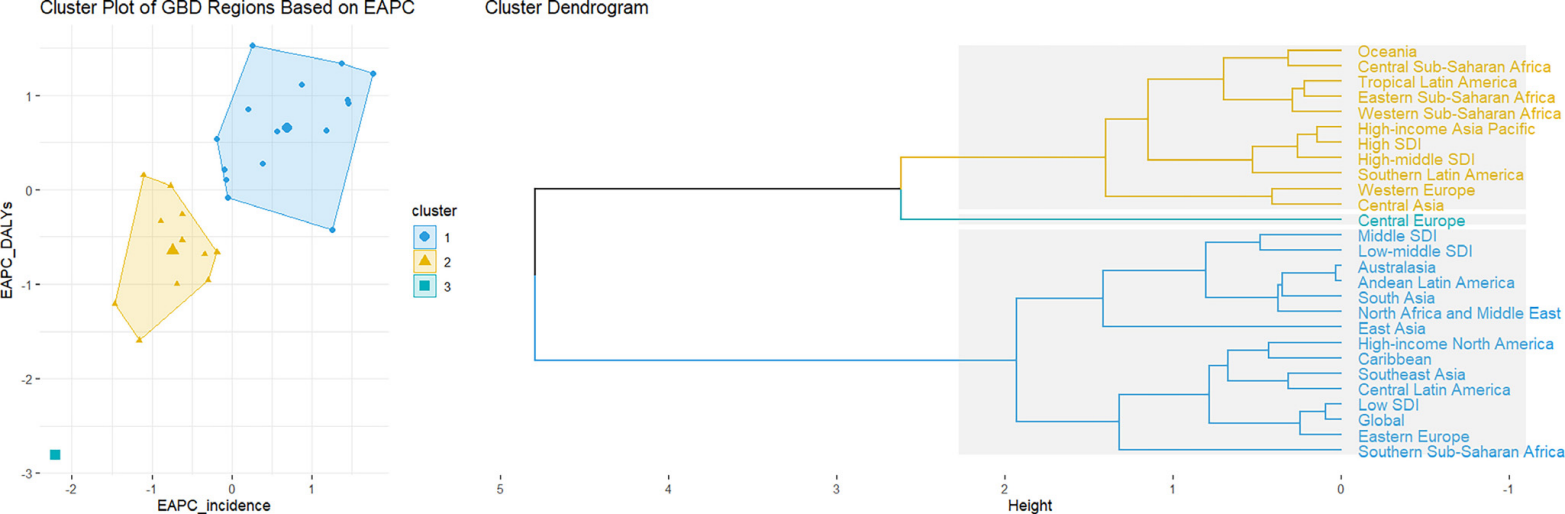
**Hierarchical clustering analysis based on EAPC.** The left panel represents a scatter plot based on EAPC clustering. The horizontal axis indicates the trend in incidence rates across different regions, while the vertical axis shows the trend in disease burden changes. Data points are grouped into three clusters, with points in the same cluster representing similar trends between 1990 and 2021. The right panel presents the corresponding hierarchical clustering dendrogram. EAPC: Estimated annual percentage change.

### Sex patterns and age

**Figure 5.**
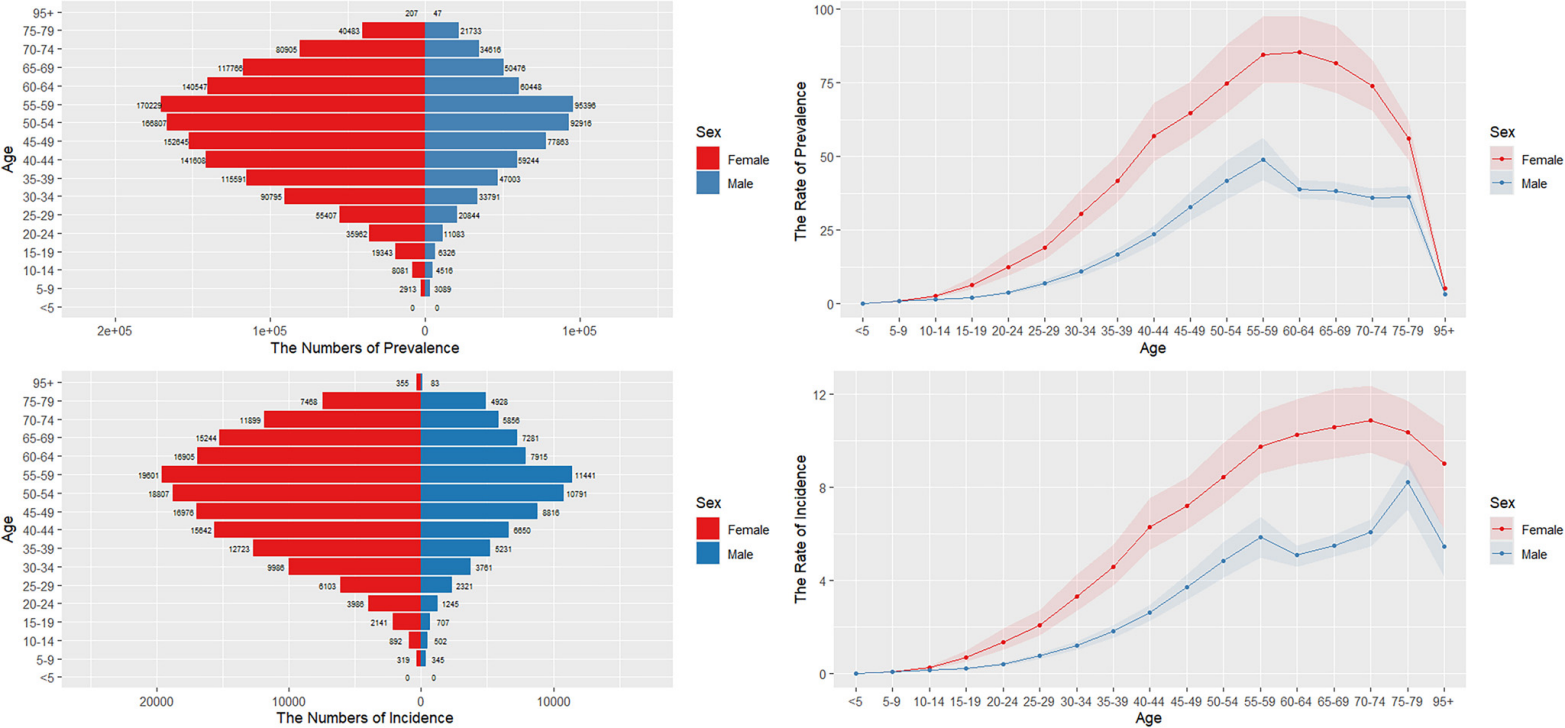
**Trend chart of TC burden stratified by age and sex**. TC: Thyroid cancer.

In 2021, the global prevalence rate for women was 31.33 per 100,000 (95% UI: 27.56–36.85), while for men it was 14.79 per 100,000 (95% UI: 12.96–16.44). The incidence rate for women was 3.83 per 100,000 (95% UI: 3.36–4.49), compared to 1.98 per 100,000 for men (95% UI: 1.72–2.19). The DALY rate for women was 16.7 per 100,000 (95% UI: 14.09–19.59), and for men it was 12.34 per 100,000 (95% UI: 10.34–13.77). The burden of disease and incidence increased with age among middle-aged and early elderly populations, peaking at ages 55–59 for both prevalence and incidence, followed by a decline. In terms of prevalence, women reached their peak at ages 60–64, while men peaked at 55–59. Regarding incidence, women peaked at 70–74 years and men at 75–79 years, with a secondary peak observed for both sexes at 55–59 years (Figure 5). The incidence of TC increased steadily over the years, with the most significant rise occurring between 2003 and 2009. After that period, the growth rate gradually stabilized (Figure 1A and 1B).

**Figure 6.**
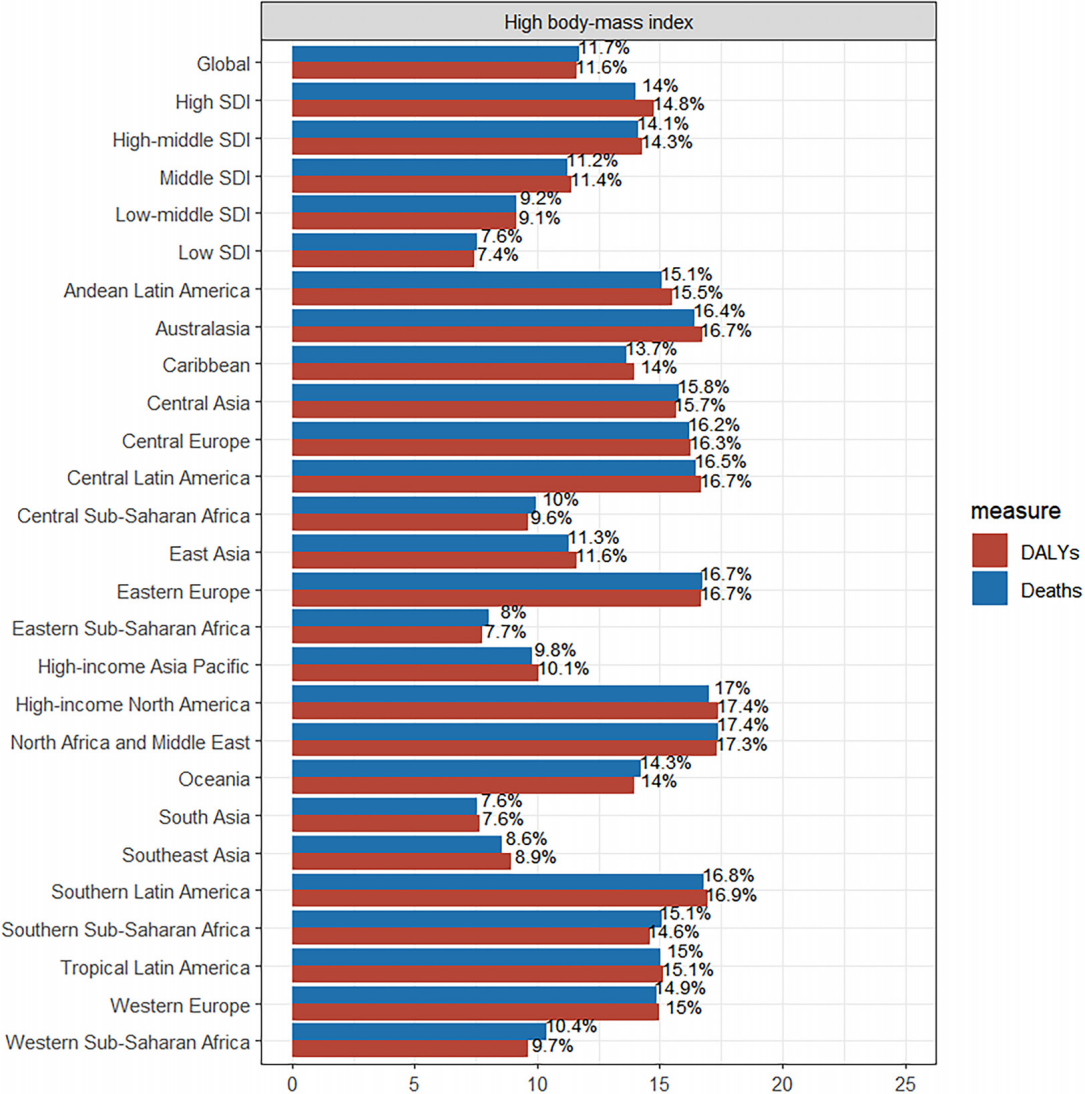
**Illustrates the major risk factors influencing DALYs**. DALYs: Disability-adjusted life years.

### Risk factors

This study identified high BMI as a major risk factor through global data analysis. The regions with the highest ASDR attributable to high BMI are North Africa and the Middle East (17.4%). The greatest impact of high BMI on DALYs is observed in high-income North America (17.4%). At the SDI stratification level, the burden of high BMI on deaths and DALYs is higher in high and high-middle SDI regions (Figure 6).

### Future projections of global TC burden

**Figure 7.**
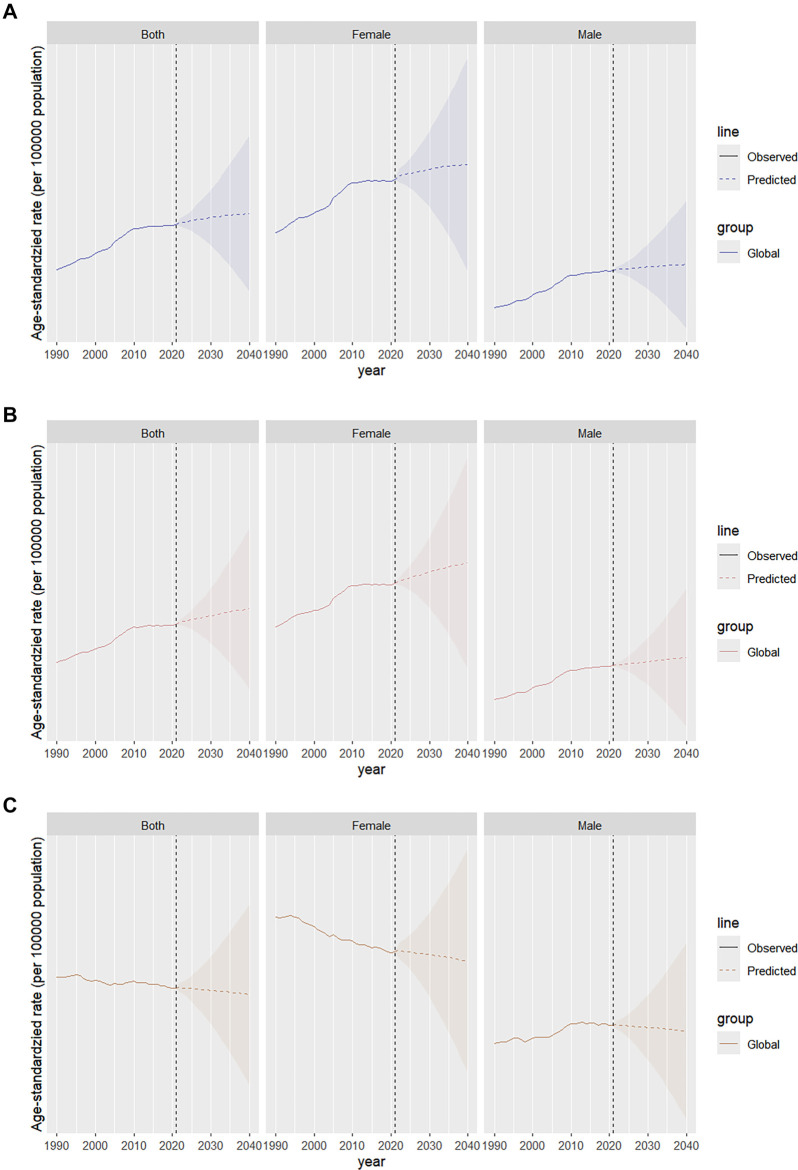
**Future projections of the global burden of TC**. TC: Thyroid cancer.

The global prevalence of TC is projected to rise from 24.06 per 100,000 in 2021 (95% UI: 24.03–24.10) to approximately 26.12 per 100,000 in 2040 (95% UI: 11.30–40.95). Among men, the rate is expected to increase to 16.51 per 100,000 (95% UI: 4.38–28.65), while among women, it is projected to rise from 32.60 per 100,000 (95% UI: 32.55–32.66) to 35.34 per 100,000 (95% UI: 15.24–55.44) (Figure 7A). The incidence of TC is also expected to increase, reaching 3.34 per 100,000 (95% UI: 1.46–5.23) by 2040. Among men, the rate is projected to rise to 2.20 per 100,000 (95% UI: 0.58–3.83), while among women, it will increase from 3.93 per 100,000 (95% UI: 3.91–3.94) to 4.40 per 100,000 (95% UI: 1.94–6.86) (Figure 7B). Meanwhile, the age-standardized DALY rate is expected to decline from 14.53 per 100,000 (95% UI: 14.51–14.56) to approximately 14.12 per 100,000 by 2040 (95% UI: 8.61–19.64). Among men, the rate is projected to decrease from 12.27 per 100,000 (95% UI: 12.24–12.31) to 11.88 per 100,000 (95% UI: 6.52–17.24), while among women, it will drop from 16.70 per 100,000 (95% UI: 16.67–16.74) to 16.15 per 100,000 (95% UI: 9.36–22.95) (Figure 7C).

## Discussion

### TC burden analysis

Advances in diagnostic technology may be contributing to the increasing global burden of TC. Additionally, heightened public awareness of health and the widespread use of general health check-ups have significantly improved TC detection rates, which may be another major factor behind the rise in epidemiological data [16]. Although the ASPR and ASIR of TC have shown an upward trend, both the mortality rate and disability-DALY rate have declined. This contrasting outcome underscores the effectiveness of expanded screening coverage and the improved diagnostic and therapeutic skills of healthcare professionals.

Compared to 1990, the increases in ASPR and ASIR in High and High-middle SDI regions in 2021 were relatively modest, while ASDR and DALY rates exhibited a downward trend. This phenomenon may be attributed to the strong early diagnostic capabilities and abundant medical resources in these regions, which are sufficient to improve patients’ quality of life [17]. In contrast, in other regions, higher mortality and DALY rates may result from the lack of effective screening and diagnostic technologies, leading to diagnoses only at advanced stages—substantially increasing the difficulty of treatment [1, 18]. Margherita Pizzato and her team have noted that the epidemiological pattern of high incidence but low mortality in TC is largely due to overdiagnosis [19]. A previous study found that, between 1988 and 2007, over 500,000 individuals in 12 high-income countries may have been overdiagnosed with TC [20]. Overdiagnosis and the resulting overtreatment have placed a significant burden on healthcare systems, and this issue has received increasing attention in recent years. In response, international guidelines have undergone substantial revisions, with clear recommendations to avoid TC screening in asymptomatic individuals [21]. In 2021, advanced medical equipment and screening practices in North America and the Asia-Pacific region improved early TC diagnosis rates, placing these regions among global leaders in TC-related epidemiological metrics. However, compared to 1990, the growth rate in these regions ranks in the middle to lower range globally, with both mortality and DALY rates trending downward [2, 22]. In contrast, regions such as the Andes, Latin America, and South Asia have seen relative increases in prevalence, morbidity, mortality, and DALYs. Factors such as increased early-warning screenings and job-related stress may contribute to higher prevalence and morbidity. Meanwhile, insufficient improvement in treatment and care capacity may explain the rise in disability rates [4]. Additionally, the region’s high altitude—averaging 2,358 meters—may also be associated with a higher incidence of TC [23]. Multiple studies have shown a higher number of female TC patients [5, 24]. Estrogen promotes the proliferation, migration, and invasion of TC cells through its receptors and influences the tumor microenvironment to accelerate TC progression [25]. Women undergo significant hormonal fluctuations during puberty, pregnancy, and menopause, which may increase thyroid cell instability. Furthermore, women are more likely to undergo frequent health screenings during these life stages, contributing to higher detection rates [26]. Expression levels or mutation frequencies of TC-related genes—such as BRAF and RAS—are also higher in females [27]. The presence of two X chromosomes may influence the regulation of certain tumor suppressor genes, thereby contributing to higher susceptibility in women [28]. Although TC incidence is lower in males, cases in men tend to be more malignant and more likely to metastasize to lymph nodes and distant organs, warranting closer clinical attention [29]. Both prevalence and incidence rates of TC show a positive correlation with age, with individuals aged 55 and older advised to be more vigilant with screening. Global population aging is also expected to contribute to this trend [30]. Overall, TC prevalence exhibits an inverted U-shape distribution, with middle-aged individuals showing a sharper increase in prevalence compared to the elderly—potentially due to overdiagnosis [31].

Elevated BMI is an independent risk factor for various chronic metabolic diseases and cancers. It is recognized as the second most common and modifiable carcinogenic factor after smoking [32]. Studies have shown that obesity-related factors—including low-grade chronic inflammation, altered cytokine levels, insulin resistance, and oxidative stress—affect the progression of TC [33]. In fact, the onset of TC is strongly influenced by a combination of factors [34], including lifestyle [35], environmental exposures such as radiation (from the Chernobyl disaster [36] or occupational sources [37]), and iodine intake [38], whether excessive [39] or deficient [40]. Additionally, a family history of TC [41] and a personal medical history [42] are also important risk factors. As research into the etiology of TC advances, an increasing number of potential pathogenic factors have been identified. These include other endocrine disorders [43], cytokine imbalances [44], smoking [45], alcohol consumption [46], dietary habits [47], psychological stress, and environmental pollution [48]. These factors may influence the epigenetic landscape of the body, altering gene expression and contributing to disease progression [49]. This underscores the importance of a multifactorial approach when analyzing interactions among these variables and highlights the need to continually refine both preventive and therapeutic strategies to improve disease management and optimize patient outcomes. The COVID-19 pandemic may have influenced the burden trends of TC observed in our study. The pathogenesis of COVID-19 involves autoimmune activation and cytokine storms. Since the thyroid is an endocrine organ particularly susceptible to autoimmune attack, it has a complex, bidirectional interaction with the virus [50]. A study from the United Arab Emirates found a significant increase in poor prognostic markers and aggressive subtypes of papillary thyroid carcinoma (PTC) in patients undergoing thyroidectomy after the pandemic, emphasizing the need to continuously update TC prevention and management strategies [51]. This study has several limitations, including variability in the quality and availability of data across sources, as well as uncertainties introduced by the assumptions and modeling techniques inherent in the GBD approach. Nevertheless, the findings represent the best estimates based on currently available evidence.

## Conclusion

The burden of TC remains significant and is steadily increasing, with notable disparities across regions, countries, and across different levels of the SDI. Improving health systems, reducing socioeconomic polarization, and strengthening early screening and prevention are all critical measures. Equally important are raising public awareness of the disease and avoiding overtreatment.

## Data Availability

The data that support the findings of this study are openly available in 2021 GBD database at https://vizhub.healthdata.org/gbd-results/.
